# Transplanted stents: a case report

**DOI:** 10.1186/s12872-020-01597-9

**Published:** 2020-06-30

**Authors:** Frieda-Maria Kainz, Stephanie Wallner, Keziban Uyanik-Uenal, Martin Andreas, Andreas Zuckermann

**Affiliations:** Department of Cardiac Surgery, Medical University Vienna, Waehringer Guertel 18-20, 1090 Vienna, Austria

**Keywords:** Transplantation, Stent, Cardiac allograft vasculopathy, Donor heart, Coronary artery disease

## Abstract

**Background:**

An optimal donor work-up to exclude preexisting conditions is recommended, but urgency and technical equipment in donor centers must be considered. We report a case of two coronary stents present in the donor heart and the related long-term outcome.

**Case presentation:**

A 59-year-old European male patient suffering from dilated cardiomyopathy with severely reduced left ventricular function and presenting with NYHA III underwent cardiac transplantation in 2004. At the one-year follow-up, during routine cardiac catheterization, two stents were found, one in the right coronary artery and one in the circumflex artery, in the patient’s transplanted heart. As no stent implantation was performed since transplantation, these were present prior to transplantation and had been transplanted without causing clinical signs. One of the stents showed in-stent restenosis, and the patient received an additional stent 7 years after transplantation. The other stent still showed a good result, and no further intervention has been required so far. The patient is currently in good clinical condition.

**Conclusion:**

This is the first case report of favorable long-term stented coronary arteries prior to transplantation. This case highlights the importance of the donor work-up and meticulous palpation of the coronary arteries during donor evaluation.

## Background

The leading cause of death in heart transplant recipients surviving the first year is cardiac allograft vasculopathy (CAV) [1]. CAV occurs in approximately 40% of patients 5 years after transplantation. Risk factors for CAV are older donor age, donor hypertension and male donor sex as well as donor-transmitted atherosclerosis [2]. Heart transplant recipients who develop significant stenosis may benefit from percutaneous coronary intervention (PCI) for revascularization. The long-term survival of recipients with severe CAV who undergo PCI is longer than that of patients unsuitable for PCI [3].

Donors with pre-existing coronary artery disease are not routinely considered for heart transplantation (HTX) to reduce the risk of CAV. The consensus statement from a collaboration between the American Society of Transplantation (AST) and the American Society of Transplant Surgeons (ASTS) from 2001 recommended coronary angiography in male donors above the age of 45 years and in female donors above the age of 50 years. Donors aged 35 to 50 years should undergo coronary angiography if they have 3 risk factors for coronary artery disease (CAD) or a history of cocaine abuse.

However, pretransplant cardiac catheterization may not be possible in every donor center. A clinical history and surgical examination of the heart during donor assessment may not exclude coronary artery disease. We report an uncommon case of transplanted stents that were overlooked during donor assessment and described in a heart transplant patient 1 year after transplant.

## Case presentation

A 59-year-old European male patient suffering from dilated cardiomyopathy with severely reduced left ventricular function and presenting with NYHA III underwent cardiac transplantation in 2004. Myocarditis turned out to be the main reason for subsequent dilated cardiomyopathy, which was a result of a tick bite and borreliosis infection 10 years prior. The patient remained stable with conservative therapy for approximately 5 years after the tick bite but needed an automatic implantable cardioverter defibrillator in 1999. Finally, he was sent to our department with worsening cardiomyopathy and increasing dyspnea. At that time, the patient had already received recurrent cycles of levosimendan therapy, and his clinical condition worsened. Comorbidities included arterial hypertension, noninsulin-dependent diabetes mellitus type II, diabetic polyneuropathy, moderate secondary pulmonary hypertension and hepatic steatosis.

In 2004, a blood group-, size- and weight-compatible donor organ was offered, and heart transplantation was performed. The patient received a donor heart from a 45-year-old male who died due to spontaneous subarachnoid bleeding. The donor had a history of smoking (less than 5 cigarettes per day). The electrocardiogram was normal, and X-rays showed minor basal dystelectasis. Echocardiography revealed mild mitral regurgitation without other pathological findings. An invasive left heart catheter was not performed at that time, although the patient was 45 years of age. No report of CAD or a previous PCI was evident at that time.

**Fig. 1:**
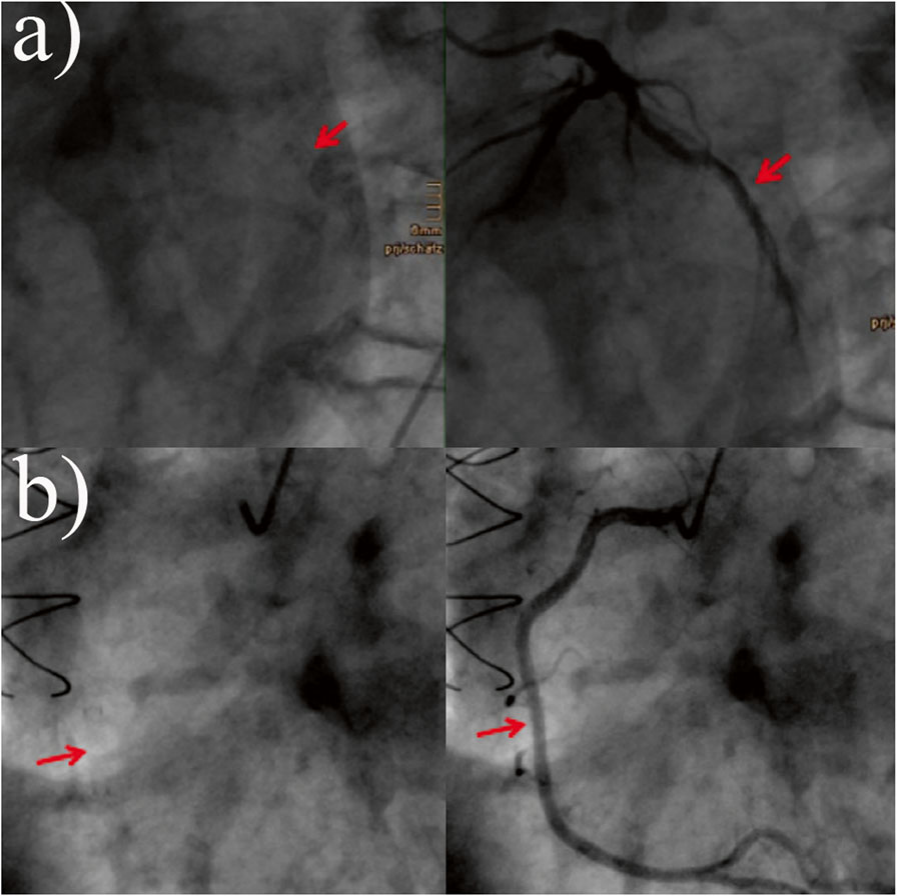
Two-year follow-up coronary angiography: **a** shows the CX stent and the filled vessel, **b** shows the RCA stent and the filled vessel

The patient successfully underwent heart transplantation without complications. The CDC crossmatch was negative. After induction therapy with anti-thymocyte globulin, the patient commenced an immunosuppression regimen of cyclosporine A in combination with mycophenolate mofetil and prednisone. He was transferred to the regular ward within 1 week and underwent the usual follow-up treatment, which included routine immunosuppressive target level controls, echocardiography, chest X-rays and endomyocardial biopsies. The patient was sent to rehabilitation after 23 days. Echocardiographic controls during the first 12 months showed normal systolic function. Our routine follow-up protocol to exclude CAV consisted of invasive coronary angiograms at 1, 3, 5, and 7 years at that time. Therefore, a coronary angiography was performed in another center for routine follow-up 1 year after transplant. Normal findings with only slight wall irregularities were reported. We analyzed the fluoroscopy data and identified the stents on this angiogram. In November 2007, a second left heart catheterization for routine control was performed. This examination revealed a post-PCI result with a stent in the circumflex coronary artery (CX) and in the right coronary artery (RCA) without relevant stenosis. Our transplant team could exclude any PCI occurring between transplantation and this finding. Therefore, the two preexisting stents had been transplanted in the donor heart (Fig. 1). The next routine angiography, 7 years posttransplant in 2011, confirmed a sustained good result for the RCA stent, but the stent in the CX exhibited a 50% in-stent restenosis. The corresponding echocardiography results still showed good left ventricular function. A follow-up angiogram 6 months later revealed a progression towards 90% stenosis in the CX. Based on that finding, PCI was performed, and the stenosis was re-stented (Xience Prime 3.0 × 15 mm, Abbott, Chicago, IL, USA). No change in immunosuppressive therapy was performed, as the patient tolerated the current protocol well and refused a change in therapy. In 2015, the last angiography confirmed a good overall vessel status with a good PCI result and no further stenosis. The patient had his last visit in our outpatient department in April 2018 and is still doing well without any restrictions.

## Discussion and conclusion

Cardiac allograft vasculopathy is one of the most important factors determining long-term survival after heart transplantation. Therefore, prevention and early diagnosis of CAV, including optimal medical management with immunosuppressive medications, is essential [4].

A global shortage of donor organs may lead to the acceptance of marginal donor organs. These may present with preexisting pathologies, which potentially increase the risk for CAV [5]. Annual or biannual coronary angiography remains the gold standard for the detection of CAV [4]. In addition, intravascular ultrasound should be considered within the first postoperative year and may identify diffuse vascular changes. However, these recommendations were published 6 years after our patient underwent transplantation, and the institutional protocol was applied for follow-up.

Revascularization procedures are restricted to a relatively small proportion of patients with approachable coronary lesions. PCI is preferred over coronary artery bypass grafting (CABG) [6]. However, CABG is feasible in selected patients, though it has limited efficiency in diffuse distal CAV. PCI is able to avoid or bridge the time to retransplantation [7, 8]. A cohort study from Goekler et al. compared PCI to conservative treatment for CAV and revealed a significantly better midterm outcome in the intervention group [9]. Drug-eluting stents (DESs) showed favorable angiographic outcomes in patients suffering from CAV [10]. Colombo et al. reported 96% primary success with DESs in 135 lesions, but 23% of patients suffered from in-stent restenosis [11]. Beygui et al. described better preservation of left ventricular function with DESs than with bare metal stents (BMSs) [12]. Compared to BMSs, DESs showed a significant reduction in in-stent restenosis in heart transplant patients [11, 12]. However, the risk of restenosis was higher than that with CAD in nontransplanted patients.

In-stent restenosis after PCI decreases long-term prognosis, aggravated by the fact that most patients remain asymptomatic. Lee et al. investigated 105 patients who underwent PCI with either a DES or a BMS [13]. In-stent restenosis occurred in 26 patients (31.3%) and was treated by target vessel revascularization in 19 patients (73.1%), whereas 3 (11.5%) patients underwent retransplantation, and 4 (15.4%) patients received medical therapy alone. Patients with in-stent restenosis showed a lower survival rate than those without (38.5% versus 84.2%). The only definitive treatment for severe CAV is retransplantation, but this procedure is limited by a shortage of donor organs and has a worse prognosis than the first transplantation. Therefore, retransplantation is reserved for selected cases.

This is the first case report of favorable long-term stented coronary arteries prior to transplantation. This highlights the importance of the donor work-up and meticulous palpation of the coronary arteries during donor evaluation. Pretransplant coronary angiography should also be performed in younger donors with specific risk factors. Furthermore, the surprisingly slow progression of coronary artery disease after transplantation may be related to an altered lifestyle of the recipient, additional medication or even a different genetic background between the recipient and donor, but this is beyond the scope of this report.

## Data Availability

Available upon request.

## Abbreviations

BMS: Bare metal stent
CABG: Coronary artery bypass grafting
CAV: Cardiac allograft vasculopathy
CX: Circumflex artery
DES: Drug-eluting stent
HTX: Heart transplantation
IVUS: Intravascular ultrasound
PCI: Percutaneous coronary intervention
RCA: Right coronary artery
